# Pathological complete response and prognostic predictive factors of neoadjuvant chemoimmunotherapy in early stage triple-negative breast cancer

**DOI:** 10.3389/fimmu.2025.1570394

**Published:** 2025-05-12

**Authors:** Xuwei Chen, Hengming Ye, Daming Xu, Siqi Chen, Wei Wu, Xiaoyu Qian, Xinyu Zhang, Xuxiazi Zou, Junquan Chen, Xi Wang

**Affiliations:** ^1^ Department of Breast Oncology, Sun Yat-sen University Cancer Center, Guangzhou, Guangdong, China; ^2^ Department of Experimental Research, State Key Laboratory of Oncology in South China, Guangzhou, Guangdong, ;China; ^3^ Department of Experimental Research, Collaborative Innovation Center for Cancer Medicine, Guangzhou, Guangdong, China; ^4^ Department of Disease Prevention and Control, Public Health Service Center of Bao'an District, Shenzhen, Guangdong, China; ^5^ Department of Pathology, Sun Yat-sen University Cancer Center, Guangzhou, Guangdong, China; ^6^ Department of Medical Oncology, Sun Yat-sen University Cancer Center, Guangzhou, Guangdong, China; ^7^ Zhongshan School of Medicine, Sun Yat-sen University, Guangzhou, China; ^8^ Department of Anesthesiology, Sun Yat-sen University Cancer Center, Guangzhou, Guangdong, China

**Keywords:** early-stage triple-negative breast cancer, neoadjuvant chemoimmunotherapy, pathological complete response, inflammatory markers, systemic inflammatory response index

## Abstract

**Background:**

Neoadjuvant chemoimmunotherapy (nCIT) has shown promise in treating early-stage triple-negative breast cancer (eTNBC), but predictive biomarkers for pathological response and prognosis remain poorly defined.

**Objective:**

This study aimed to explore pathological complete response and prognostic predictive factors in eTNBC patients treated with nCIT.

**Materials and methods:**

We retrospectively analyzed 112 eTNBC patients who underwent surgery after nCIT at Sun Yat-sen University Cancer Center between June 2019 and June 2023. Pathological response was assessed using Miller-Payne grade. Clinicopathological features and hematologic markers were analyzed with univariate and multivariate logistic regression or Cox regression, as well as Kaplan-Meier survival curves. Objective response rate (ORR), pathological complete response (pCR), and disease-free survival (DFS) were evaluated. Nomograms predicting pCR and DFS were constructed based on significant risk factors and the systemic inflammatory response index (SIRI).

**Results:**

Higher baseline lymphocyte counts (*P*=0.004) were independently associated with a higher pCR rate, while elevated monocyte counts (*P*=0.006), neutrophil-to-lymphocyte ratio (*P*=0.005), platelet-to-lymphocyte ratio (p = 0.005), SIRI (*P*=0.037), systemic immune-inflammation index (*P*=0.029), and preoperative SIRI (*P*=0.010) were associated with a lower pCR rate. Higher baseline SIRI (*P*= 0.009) was correlated with shorter DFS, while higher preoperative lymphocyte counts (*P*=0.019) predicted longer DFS. Nomograms incorporating SIRI showed high accuracy in predicting pCR and DFS.

**Conclusion:**

Hematologic inflammatory markers, particularly SIRI, are cost-effective and reliable predictors of prognosis and treatment efficacy in eTNBC patients undergoing nCIT, helping clinicians develop personalized treatment strategies.

**Clinical trial registration:**

https://www.medicalresearch.org.cn/, identifier MR-44-24-046099.

## 1 Introduction

Breast cancer (BC) is the second commonly diagnosed malignancy worldwide and a leading cause of cancer-related mortality among women (1). Neoadjuvant chemotherapy (NACT) is an essential component of curative-intent strategies for patients with early-stage triple-negative breast cancer (eTNBC) (2, 3). Patients who achieve a pathological complete response (pCR) following neoadjuvant chemotherapy (NACT) demonstrate improved outcomes, including significantly longer event-free survival (EFS) and overall survival (OS) compared to those who do not (4–6). This underscores the importance of achieving a pCR for better long-term outcomes. Although NACT has shown significant efficacy, therapeutic outcomes vary considerably among patients. Approximately 35% to 40% of patients achieve pCR, leaving a substantial proportion who do not respond adequately, resulting in worse outcomes (4, 7). This moderate respond rate highlights an urgent need for innovative therapeutic strategies to enhance treatment efficacy and improve patient outcomes.

Recent advancements in neoadjuvant therapy, particularly the combination of immunotherapy with chemotherapy, have significantly improved pCR rates, EFS, and OS in patients with eTNBC (8–10). This combination offers a promising new strategy in the treatment landscape for eTNBC patients. Nevertheless, it is important to note that not all individuals respond favorably to neoadjuvant chemoimmunotherapy (nCIT), approximately 36% of patients fail to achieve pCR and 20.3% of patients recurred or metastasized after treatment (8, 11). To maximize therapeutic efficacy and minimize toxicity, thereby enabling the implementation of more precise and personalized treatment strategies and optimizing outcomes while reducing unnecessary adverse effects, it is essential to accurately predict which patients are most likely to benefit from nCIT. Although programmed death-ligand 1 (PD-L1) expression and tumor-infiltrating lymphocytes (TILs) are considered potential predictive biomarkers for neoadjuvant immunotherapy efficacy, the absence of standardized PD-L1 diagnostic criteria and the limited clinical implementation of TILs assessment currently restrict their overall utility (12–15). Hence, there is an urgent need for a cost-effective, efficient, and easily accessible biomarker to predict the efficacy of nCIT, enabling the identification of patients more likely to benefit from this therapy (16).

Systemic inflammation is a critical factor in tumorigenesis and cancer progression, impacting treatment efficacy and disease outcomes. It drives angiogenesis, promotes malignant transformation, and creates an immunosuppressive microenvironment, thereby facilitating cancer cell proliferation and metastasis (17, 18). Hematologic-related inflammation markers, including neutrophil-to-lymphocyte ratio (NLR), derived NLR (dNLR), platelet-to-lymphocyte ratio (PLR), systemic inflammatory response index (SIRI), and systemic immune-inflammation index (SII), have been investigated as economical and practical biomarkers with significant utility in predicting pathological responses to NACT in eTNBC (19–25), highlighting its potential as a biomarker for forecasting treatment outcomes and prognosis. However, the predictive value of these readily accessible hematologic markers in patients with eTNBC receiving nCIT remain unclear.

This retrospective study aimed to evaluate the association between hematologic inflammatory markers and the pathological response and prognosis of eTNBC patients treated with neoadjuvant chemoimmunotherapy.

## 2 Materials and methods

### 2.1 Study design and patient selection

**Figure 1:**
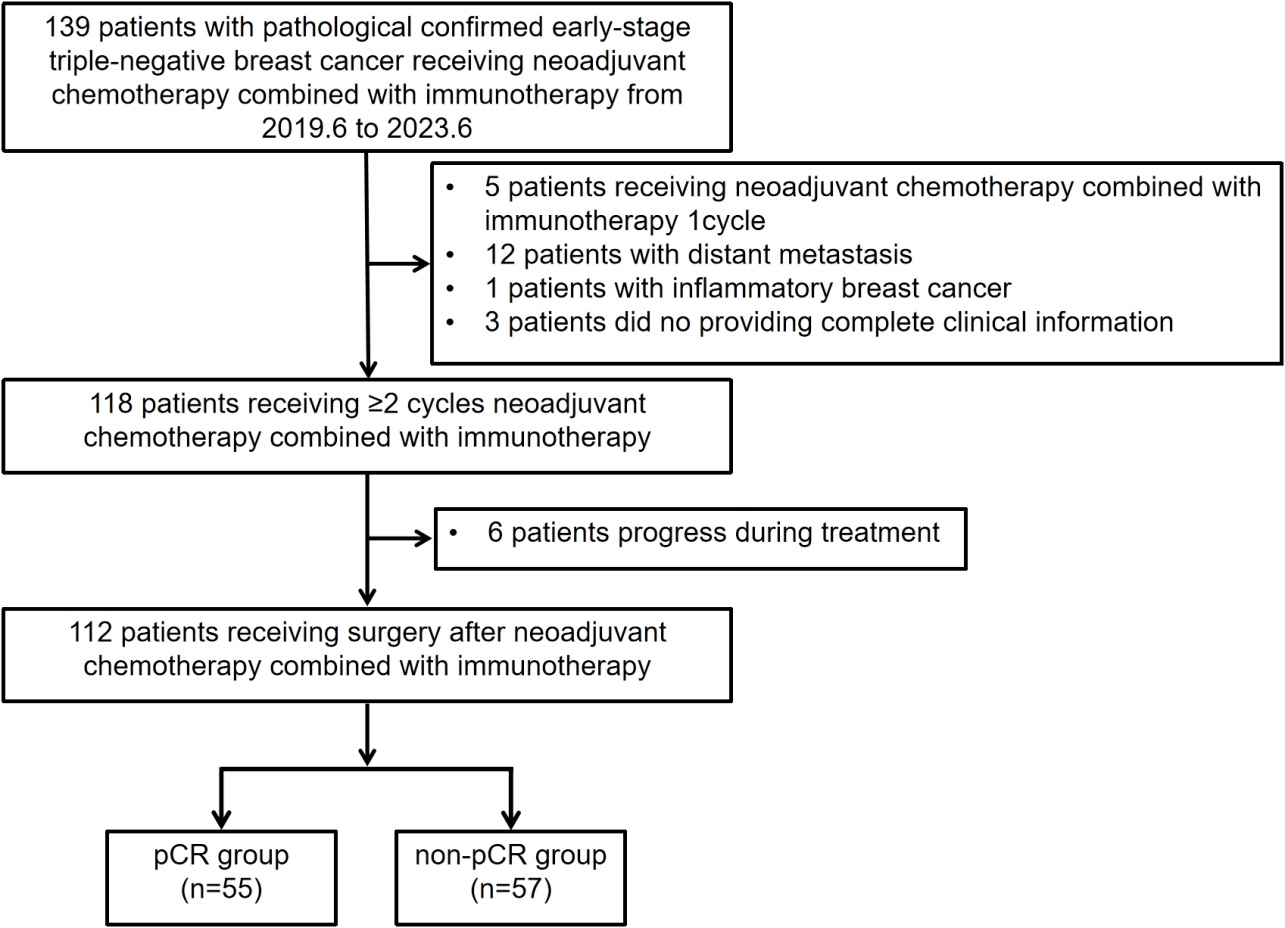
Flowchart illustrating the eligibility and inclusion process of patients with early stage triple-negative breast cancer receiving neoadjuvant chemotherapy combined with immunotherapy.

This was a single-center, retrospective cohort study. Patients with early stage, resectable eTNBC who underwent nCIT followed by curative surgery at Sun Yat-sen University Cancer Center from June 2019 to June 2023 were retrospectively reviewed. The inclusion criteria were as follows (1): histopathological diagnosis of clinical stage IIA–IIIC eTNBC (2); received two or more cycles of nCIT (3); underwent radical surgery (4); availability of complete clinical data. The exclusion criteria included (1): receipt of only one cycle of nCIT (2); diagnosed with distant metastasis (3); diagnosed with inflammatory BC (4); progression or recurrence during neoadjuvant treatment. A total of 112 patients from our cancer center were enrolled in this study. **Figure 1** presents a flowchart summarizing the patient inclusion process.

### 2.2 Data extraction and assessment

All enrolled patients underwent standard pre-treatment examinations in accordance with National Comprehensive Cancer Network (NCCN) guidelines. These included pre-treatment tumor biopsy, breast mammography, breast ultrasound, contrast-enhanced chest computed tomography (CT), and positron emission tomography/CT. Clinical and pathological staging followed the eighth edition of the TNM staging system for BC. Consistent with international practice, all patients received 2 to 9 cycles of intravenous nCIT, administered on the first day of each three-week cycle. Clinicopathologic features collected included age, body mass index (BMI, weight in kilograms divided by height in meters squared), menopausal status, clinical and pathological TNM stage, immune checkpoint inhibitors used, neoadjuvant immunotherapy dosage, surgical approach, hematological parameters, and Miller-Payne grade.

Peripheral blood samples were collected within one week before the first chemoimmunotherapy and within one week before surgery. The neutrophil-to-lymphocyte ratio (NLR), derived NLR (dNLR), platelet-to-lymphocyte ratio (PLR), systemic inflammatory response index (SIRI), and systemic immune-inflammation index (SII) were calculated as follows (18): NLR = neutrophils/lymphocytes, dNLR = neutrophils/(leucocytes - neutrophils), PLR = platelets/lymphocytes, SIRI = neutrophils × monocytes/lymphocytes, SII = platelets × neutrophils/lymphocytes.

### 2.3 Pathologic evaluation

Pathological response was evaluated using the Miller-Payne grade after nCIT, with responses classified as complete response (CR), partial response (PR), stable disease (SD), or progressive disease (PD). Lymph node involvement and lymphovascular invasion (LVI) were assessed, along with necrosis and stromal changes. Pathological complete response (pCR) was defined as the absence of viable tumor cells in the residual primary tumor and lymph nodes. All pathological assessments were performed independently by two pathologists, each with more than 10 years of experience; disagreements were resolved by consensus. The pathological efficacy of nCIT on primary breast lesions was assessed using the Miller-Payne grading system, which ranges from 1 to 5 (26).

The expression levels of estrogen receptor (ER), progesterone receptor (PR), human epidermal growth factor receptor 2 (HER-2), and Ki-67 were determined by immunohistochemistry. Hormone receptor-positive (HR+) expression was defined as 1% or more cells stained for ER or PR. HER-2 status was evaluated according to recent guidelines (27). A Ki-67 index greater than 30% was defined as high expression, based on the latest guideline (28). In accordance with ESMO diagnostic criteria (29), TNBC was characterized by dual immunohistochemical parameters (1): ER and PR expression in <10% of tumor cells, and (2) HER2 negativity defined as immunohistochemical (IHC) scores of 0, 1+, or 2+ without gene amplification confirmed by *in situ* hybridization.

### 2.4 Endpoints and follow-up

The primary endpoint was pCR. The secondary endpoint was disease-free survival (DFS), defined as the time from surgery to the first recurrence, progression, or last follow-up (April 2024). Follow-up data were collected through electronic medical records, institutional databases, and phone calls.

### 2.5 Statistical analysis

Based on the assessment of pathological responses after surgery, patients were divided into two groups: pCR (n = 55) and non-pCR (n = 57). Differences in demographic, clinical, and hematologic characteristics between these two groups were analyzed. Categorical variables were analyzed using the χ² test. The Shapiro-Wilk test was used to evaluate normal distribution. Normally distributed continuous variables were expressed as mean ± standard deviations, and comparisons were made using the t-test. Continuous variables with non-normal distributions were expressed as medians and interquartile range (IQR) and analyzed using the Mann-Whitney U test. The optimal cut-off value of hematologic markers for predicting non-pCR was determined using ROC curve analysis and Youden index (**Supplementary Table S1**). Univariate and multivariate logistic regression or Cox regression analyses were performed to identify the effects of clinicopathological features on pCR and DFS. Variables adjusted in the multivariable logistic regression or Cox regression models were determined based on previous studies and the factors known to affect breast cancer prognosis. These included age at diagnosis group, BMI group, T grade, N grade, pretreatment subtypes, chemotherapy regimens, and doses of neoadjuvant therapy. The Kaplan-Meier estimator was used to calculate and compare DFS. Nomograms predicting the probability of pCR and DFS were developed based on optimal predictive models incorporating independent risk factors. The performance of the models were assessed for internal validation by the Harrell’s concordance index (C-index) and calibration curves using bootstrapping (random sampling with replacement) 1000 times. To interpret the contribution of individual clinical variables to model predictions of pCR and DFS, SHapley Additive exPlanations (SHAP) analysis was performed. SHAP values were computed using the fastshap package in R (version 4.3.3), which provides efficient estimation of feature contributions for black-box models. Visualization of the SHAP values was conducted using the shapviz package, generating both summary plots and bar plots to display the distribution and average importance of each feature. All statistical analyses were performed using SPSS software (version 27.0). A p-value of less than 0.05 was considered significant for all analyses.

## 3 Results

### 3.1 General clinical characteristic, pathological response and morphological changes

A total of 112 patients were enrolled and divided into pCR (n=55) and non-pCR (n=57) groups. All patients underwent R0 resections. At initial pre-treatment clinic staging, the majority of patients were classified as T2 (n=74, 66.1%) and N1 (n=58, 51.8%), with clinical stages IIB (n=45, 40.2%) and III (IIIA, 17.8%; IIIC, 18.8%) comprising the largest portions of the cohort. There were no statistical differences between the cohorts regarding age, BMI, menopausal status, family history, Ki-67 index, pretreatment subtypes, or use of immune checkpoint inhibitors (**Table 1**).

**Table 1:** Demographic and clinicopathological characteristics of all patients.

Characteristics	Total (n=112,%)	pCR (n=55,%)	Non-pCR (n=57,%)	*P*
Age, years (Mean±SD)	47.47±10.37	47.59±10.01	47.35±10.79	0.903
BMI, kg/m^2^ (Mean±SD)	23.33±3.06	22.95±3.17	23.70±2.93	0.207
Menopausal status (pre-menopausal/post-menopausal)	76 (67.9)/36 (32.1)	40 (72.7)/15 (27.3)	36 (63.2)/21 (36.8)	0.278
Family history (No/Yes)	90 (80.4)/22 (19.6)	45 (81.8)/10 (18.2)	45 (78.9)/12 (21.1)	0.702
T grade (1/2/3/4)	12 (10.7)/74 (66.1)/25 (22.3)/1 (0.9)	8 (14.5)/40 (72.7)/7 (12.7)/0 (0.0)	4 (7.0)/34 (59.6)/18 (31.6)/1 (1.8)	0.054
N grade (0/1/2/3)	20 (17.9)/58 (51.8)/13 (11.6)/21 (18.8)	10 (18.2)/37 (67.3)/2 (3.6)/6 (10.9)	10 (17.5)/21 (36.8)/11 (19.3)/15 (26.3)	0.002
Clinical stage (IIA/IIB/IIIA/IIIC)	26 (23.2)/45 (40.2)/20 (17.8)/21 (18.8)	11 (27.3)/30 (54.5)/4 (7.3)/6 (10.9)	11 (19.3)/15 (26.3)/16 (28.1)/15 (26.3)	0.001
Ki-67 index (≤30%/>30%)	13 (11.6)/98 (87.5)	4 (7.3)/51 (92.7)	9 (16.1)/47 (83.9)	0.149
Immune checkpoint inhibitors (camre/pem/sinti/tisle/tori)	8 (7.1)/24 (21.4)/14 (12.5)/43 (38.4)/23 (20.5)	0 (0.0)/13 (23.6)/7 (12.7)/23 (41.8)/12 (21.8)	8 (14.0)/11 (19.3)/7 (12.3)/20 (35.1)/11 (19.3)	0.078
Chemotherapy regimens (EC or AC/TCb/TCb-EC or AC/Others)	20 (17.9)/40 (35.7)/38 (33.9)/14 (12.5)	4 (7.3)/22 (40.0)/25 (45.5)/4 (7.3)	16 (28.1)/18 (31.6)/13 (22.8)/10 (17.5)	0.003
Doses of nCIT (2~4/5~6/7~9)	39 (34.8)/30 (26.8)/43 (38.4)	12 (21.8)/19 (34.5)/24 (43.6)	28 (47.4)/11 (19.3)/19 (33.3)	0.015
Surgery type (Mastectomy/Breast conserving surgery)	23 (20.5)/89 (79.5)	13 (23.6)/42 (76.4)	10 (17.5)/47 (82.5)	0.425
Lymph node dissection (SLNB/ALND)	10 (8.9)/102 (91.1)	3 (5.5)/52 (94.5)	7 (12.3)/50 (87.7)	0.205
Lymphovascular invasion (No/Yes)	92 (82.1)/20 (17.9)	55 (100.0)/0 (0.0)	37 (64.9)/20 (36.2)	<0.001
Radiologic response assessment (CR/PR/SD/PD)	55 (49.1)/46 (41.1)/10 (8.9)/1 (0.9)	55 (100.0)/0 (0.0)/0 (0.0)/0 (0.0)	0 (0)/46(80.7)/10 (17.5)/1 (1.8)	<0.001
Objective response rate (CR+PR)	101(90.2)	55 (100.0)	46(80.7)	<0.001
Disease control rate (CR+PR+SD)	111(99.1)	55 (100.0)	56(98.2)	1.000
Miller payne grade (1~4/5)	56 (50.0)/56 (50.0)	0 (0.0)/55 (0.0)	56 (98.2)/1 (1.8)	<0.001
ypT (0/1/2/3)	57 (50.9)/32 (28.6)/17 (15.2)/3 (2.7)	55 (100.0)/0 (0.0)/0 (0.0)/0 (0.0)	2 (3.7)/32 (59.3)/17 (31.5)/3 (5.6)	<0.001
ypN (0/1/2/3)	79 (70.5)/17 (15.2)/11 (9.8)/3 (2.7)	55 (100.0)/0 (0.0)/0 (0.0)/0 (0.0)	24 (43.6)/17 (30.9)/11 (20.0)/3 (5.5)	<0.001
Progression (No/Yes)	99 (88.4)/13 (11.6)	55 (100.0)/0 (0.0)	44 (77.2)/13 (22.8)	<0.001
DFS, months (Median, IQR)	13.00 (7.25-23.00)	14.00 (8.00-23.00)	11.00 (7.00-25.00)	0.643

pCR, pathological complete response; camre, camrelizumab; pem, pembrolizumab; sinti, sintilimab; tisle, tislelizumab; tori, toripalimab; nCIT,neoadjuvant immunochemotherapy;SLNB, sentinel lymph node biopsy; ALND, axillary lymph node dissection; CR, complete response; PR, partial response; SD, stable disease; PD, progressive disease; DFS, disease-free survival; IQR, interquartile range.

**Figure 2:**
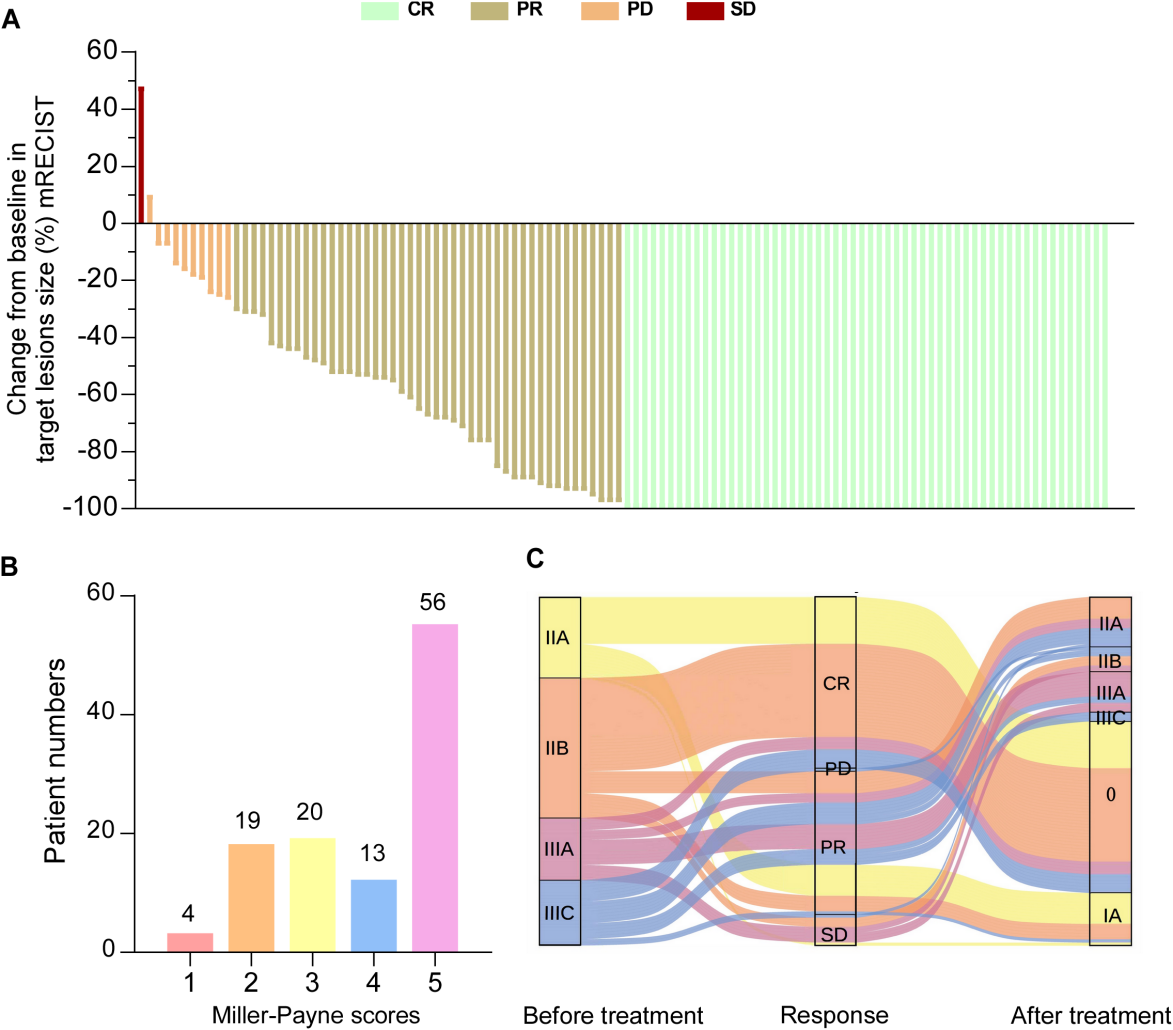
Pathological responses to neoadjuvant chemoimmunotherapy (nCIT) in patients with early-stage triple-negative breast cancer **(A)** Waterfall plot illustrating the response categories (CR/PR/SD/PD) according to RECIST 1.1 criteria. **(B)** Miller-Payne grades of breast cancer patients undergoing nCIT. **(C)** Changes in clinical stage before and after nCIT of all patients. CR, complete response; PR, partial response; SD, stable disease; PD, progressive disease; RECIST, response evaluation criteria in solid tumors.

Postoperative pathological response assessment showed that 55 patients (49.1%) achieved pCR, while 46 patients (41.1%) had a partial response (PR), 10 patients (8.9%) had stable disease (SD) and 1 patient (0.9%) had progressive disease (PD), resulting in an objective response rate (ORR) of 90.2% (*P* < 0.001) (**Table 1**, **Figure 2A**). After nCIT, the majority of patients were classified as ypT0 (n = 57, 50.9%) and ypN0 (n=79, 70.5%). Fifty-six patients (50.0%) were evaluated as Miller-Payne grade 5 (**Figure 2B**). Forty patients in the non-pCR group exhibited downstaging following nCIT (**Figure 2C**).

**Figure 3:**
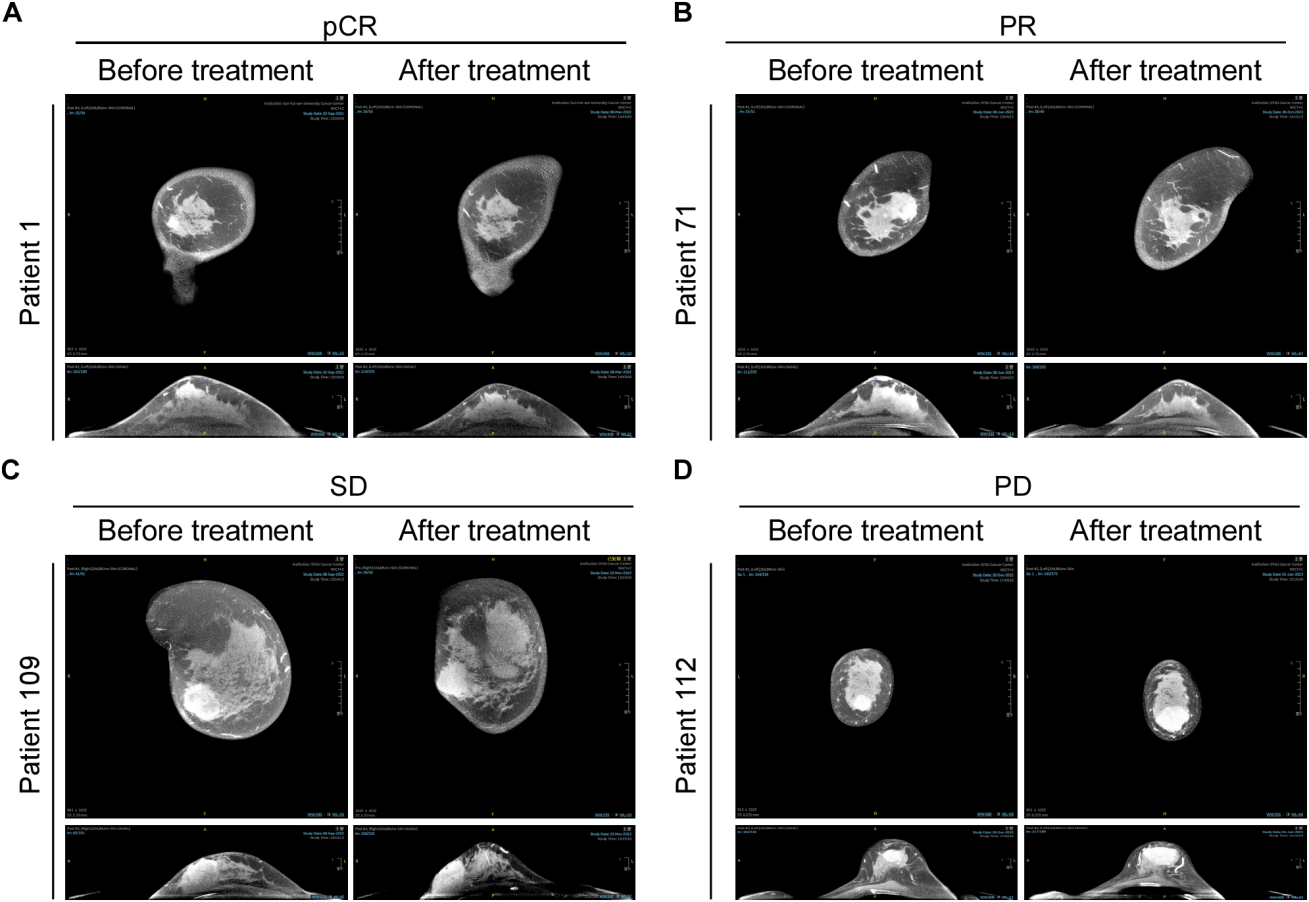
CT Images of representative patients before and after neoadjuvant chemoimmunotherapy (nCIT), demonstrating radiological responses according to RECIST 1.1 Criteria **(A–D)** Representative CT images showing pathological complete response (pCR), partial response (PR), stable disease (SD), and progressive disease (PD) in early-stage triple-negative breast cancer patients before and after treatment.

**Figure 4:**
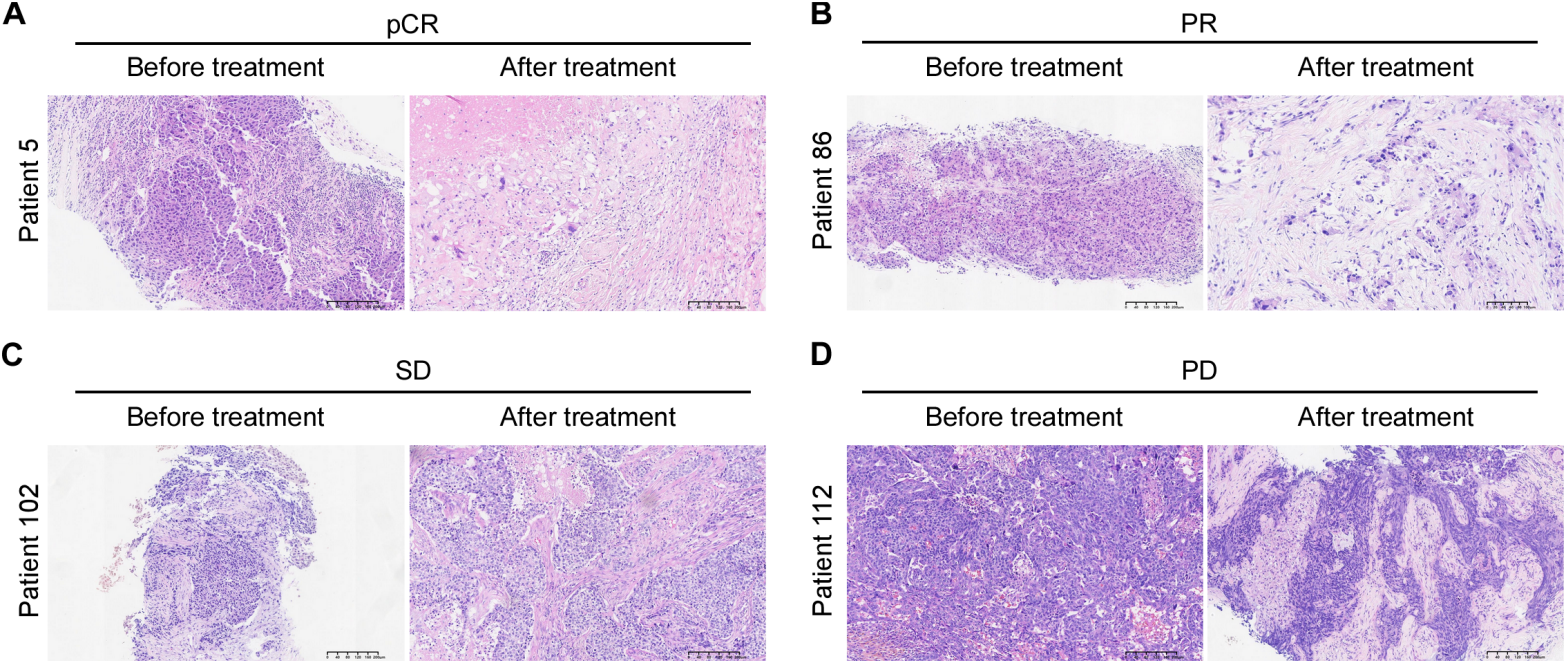
Hematoxylin and Eosin (H&E) Images of representative patients before and after neoadjuvant chemoimmunotherapy showing pathological responses according to Miller-Payne grade **(A–D)** Representative H&E images illustrating pathological complete response (pCR), partial response (PR), stable disease (SD), and progressive disease (PD) in early-stage triple-negative breast cancer patients before and after treatment.

Representative images of radiographic and pathological responses, both before and after nCIT, are shown in **Figures 3** and **4**. Morphological changes indicative of tumor regression in eTNBC after nCIT included necrosis, interstitial fibrosis, multinucleated giant cells, cholesterol crystallization, hemosiderosis, calcifications, histiocytes, lymphocytes, and other chronic inflammatory infiltrates (**Figure 4**).

Moreover, among the 13 patients who experienced disease progression during follow-up, 8 (61.5%) patients had achieved PR, 4 (30.8%) had SD, and 1 (7.7%) had PD. Ten patients developed distant metastases, including four with liver metastases, three with lung metastases, two with bone metastases, and one with contralateral upper arm skin metastasis (**Supplementary Table S2**).

### 3.2 Neoadjuvant chemoimmunotherapy regimens and hematologic parameters

Chemotherapy regimens included epirubicin plus cyclophosphamide (EC), adriamycin plus cyclophosphamide (AC), paclitaxel (T) plus carboplatin (Cb), and TCb followed by EC or AC. Tislelizumab (38.4%), pembrolizumab (21.4%) and toripalimab (20.5%) were the most commonly used immune checkpoint inhibitors. Thirty-nine patients (34.8%) received two to four cycles of nCIT, 30 patients (26.8%) received five to six cycles, and 43 patients (38.4%) received seven to nine cycles (**Table 1**).

The pCR group exhibited lower baseline monocyte count, NLR, PLR, SIRI, and SII compared to the non-pCR group. No significant differences were detected between the two groups regarding other inflammatory markers (**Supplementary Table S3**).

### 3.3 Association between Hematologic parameters and non-pCR

In univariate logistic regression, baseline platelet (*P*=0.019), baseline neutrophil (*P*=0.009), baseline lymphocyte (*P*=0.004), baseline monocyte (*P*=0.009), baseline NLR (*P*=0.001), baseline dNLR (*P*=0.009), baseline PLR (*P* < 0.001), baseline SIRI (*P*=0.003), baseline SII (*P*=0.001), preoperative PLR (*P*=0.036), preoperative SIRI (*P*=0.024) were significantly associated with a incidence of pathological response. Multivariate logistics analysis further showed that higher baseline lymphocyte counts [odds ratio (OR), 0.08; 95% CI, 0.02~0.45; *P*=0.004] and higher monocyte counts [OR, 5.46; 95% CI, 1.62~18.43; *P*=0.006], higher baseline neutrophil-to-lymphocyte ratio (NLR) [OR, 4.65; 95% CI, 1.57~13.77; *P*=0.005], higher baseline platelet-to-lymphocyte ratio (PLR) [OR, 5.95; 95% CI, 1.70~20.89; *P*=0.005], and higher baseline systemic inflammation response index (SIRI) [OR, 4.77; 95% CI, 1.10~20.78; *P*=0.037], baseline systemic immune-inflammation index (SII) [OR, 3.40; 95% CI, 1.14~10.20; *P*=0.029] and preoperative SIRI [OR, 7.45; 95% CI, 1.6~34.65; *P*=0.010] were independent risk factors associated with pathological response (**Table 2**, **Supplementary Table S4**).

**Table 2:** Univariate and multivariate analyses for non-pCR.

Characteristics	Univariate analysis		Multivariate analysis	
	OR (95%CI)	*P*	OR (95%CI)	*P*
T grade				
T2 vs. T1	1.70 (0.47, 6.14)	0.418		
T3 vs. T1	5.14 (1.17, 22.69)	0.031		
Clinical stage				
IIB vs. IIA	0.68 (0.25, 1.84)	0.450		
IIIA vs. IIA	5.46 (1.42, 20.91)	0.013		
IIIC vs. IIA	3.41 (1.00, 11.61)	0.050		
Chemotherapy regimens				
TCb vs. EC/AC	0.21 (0.06, 0.72)	0.014		
TCb-EC/AC vs. EC/AC	0.13 (0.04, 0.47)	0.002		
Others vs. EC/AC	0.63 (0.13, 3.08)	0.564		
Doses of nCIT				
5~6 vs. 2~4	0.26 (0.09, 0.70)	0.008		
7~9 vs. 2~4	0.35 (0.14, 0.87)	0.024		
Baseline platelet (>213 vs. ≤213)	3.24 (1.22, 8.63)	0.019		
Baseline neutrophil (>6.015 vs. ≤6.015)	5.69 (1.53, 21.17)	0.009		
Baseline lymphocyte (>1.165 vs. ≤1.165)	0.15 (0.04, 0.54)	0.004	0.08 (0.02, 0.45)	0.004
Baseline monocyte (>0.31 vs. ≤0.31)	3.19 (1.33, 7.67)	0.009	5.46 (1.62, 18.43)	0.006
Baseline NLR (>2.71 vs. ≤2.71)	3.68 (1.66, 8.15)	0.001	4.65 (1.57, 13.77)	0.005
Baseline dNLR (>-5.26 vs. ≤-5.26)	0.18 (0.05, 0.65)	0.009		
Baseline PLR (>140.24 vs. ≤140.24)	5.25 (2.08, 13.21)	<0.001	5.95 (1.70, 20.89)	0.005
Baseline SIRI (>2.03 vs. ≤2.03)	5.07 (1.73, 14.87)	0.003	4.77 (1.10, 20.78)	0.037
Baseline SII (>773.33 vs. ≤773.33)	3.74 (1.68, 8.33)	0.001	3.40 (1.14, 10.20)	0.029
Preoperative lymphocyte (>1.175 vs. ≤1.175)	0.52 (0.25, 1.11)	0.092		
Preoperative PLR (>242.118 vs. ≤242.118)	2.71 (1.07, 6.91)	0.036		
Preoperative SIRI (>0.403 vs. ≤0.403)	3.55 (1.18, 10.67)	0.024	7.45 (1.6, 34.65)	0.010

OR, odds ratio; NLR, neutrophilto-lymphocyte ratio; dNLR, derived neutrophil-to-lymphocyte ratio; PLR, platelet-to-lymphocyte ratio; SIRI, systemic inflammatory response index; SII, systemic immune-inflammation index.

### 3.4 Association of hematologic parameters and DFS

**Figure 5:**
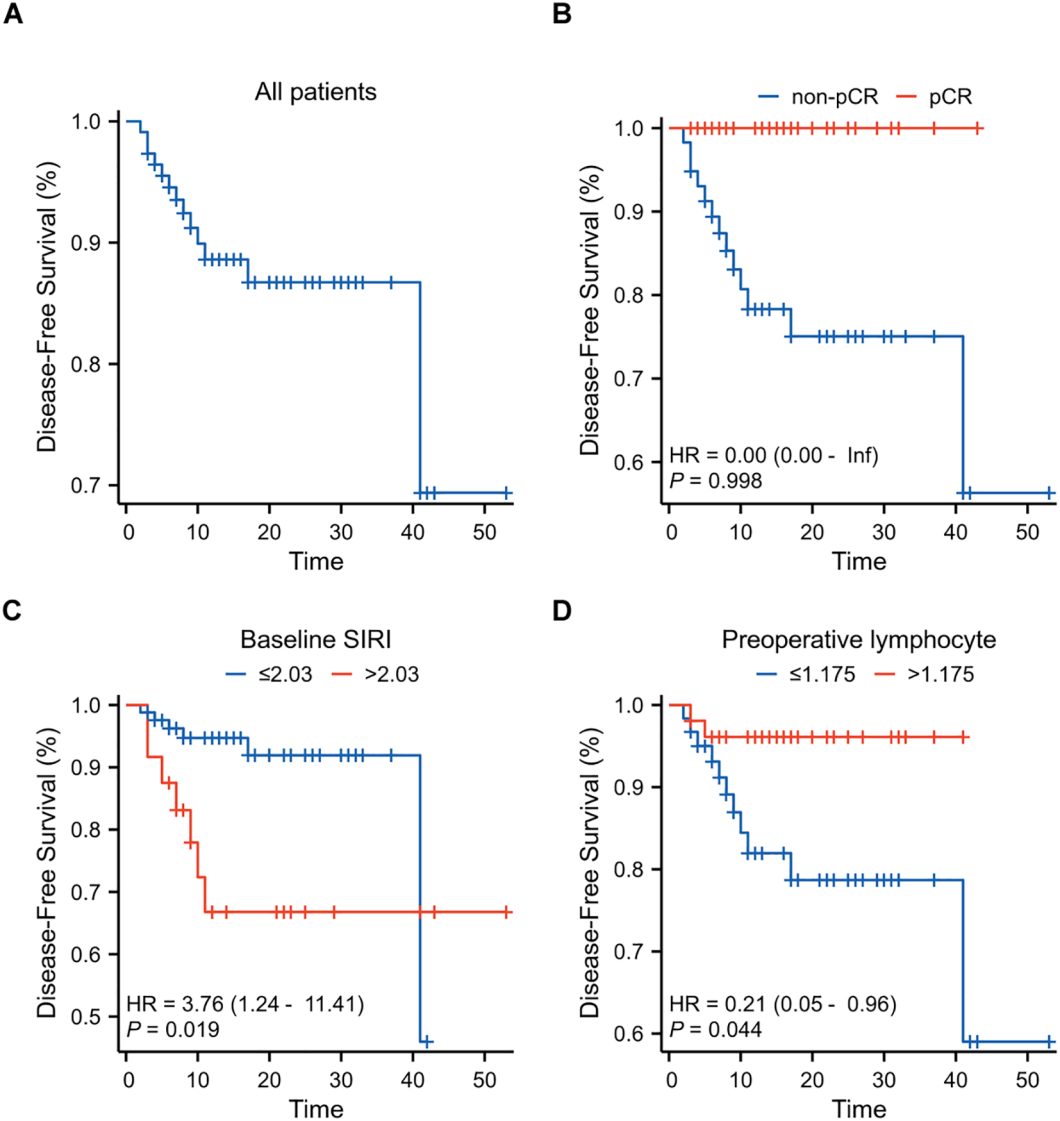
Kaplan-Meier Curves for Disease-Free Survival (DFS) in breast cancer patients after neoadjuvant chemoimmunotherapy (nCIT) **(A)** DFS rates in all patients (N=112) with early-stage triple-negative breast cancer after nCIT. **(B)** DFS rates in patients achieving pCR compared to those with non-pCR after nCIT. **(C)** Prognostic value of baseline systemic inflammatory response index (SIRI) for DFS. **(D)** Prognostic value of preoperative lymphocyte levels for DFS.

The median follow-up period was 13 months (IQR:7.25~23.00). Two patients experienced chest wall relapse, and eleven patients developed distant metastasis (**Supplementary Table S2**). Univariate Cox regression analysis showed that a higher baseline SIRI (*P=*0.019) and a higher preoperative lymphocyte (*P=*0.041) were associated with a shorter DFS. Multivariate Cox regression analysis further showed that baseline SIRI [hazard ratio (HR), 15.13; 95% CI, 1.96~116.92; *P=*0.009] and preoperative lymphocyte [HR, 0.06; 95% CI, 0.01~0.63;*P=*0.019] were independent risk factor for DFS (**Table 3**, **Supplementary Table S5**). Patients with lower baseline SIRI and higher preoperative lymphocyte counts had a better prognosis after nCIT. Kaplan-Meier curves are presented in **Figure 5**.

**Table 3:** Univariate and multivariate analyses for DFS.

Characteristics	Univariate analysis		Multivariate analysis	
	HR(95% CI)	*P* value	HR(95% CI)	*P* value
T grade				
T3~4 vs. T1~2	3.90 (1.30, 11.68)	0.015		
N grade				
N2~3 vs. N0~1	5.12 (1.57, 16.67)	0.007		
Clinical stage				
IIIA~IIIC vs. IIA~IIB	9.89 (2.19,44.66)	0.003		
Chemotherapy regimens				
TCb vs. EC/AC	0.62 (0.17, 2.30)	0.471		
TCb-EC/AC vs. EC/AC	0.14 (0.02, 1.29)	0.083		
Others vs. EC/AC	0.91 (0.20, 4.06)	0.897		
Lymphovascular invasion (Yes vs. No)	7.29 (2.36, 22.50)	0.001		
Baseline neutrophil (>6.015 vs. ≤6.015)	2.98 (0.96, 9.26)	0.059		
Baseline dNLR (>-5.26 vs. ≤-5.26)	0.34 (0.11, 1.04)	0.059		
Baseline SIRI (>2.03 vs. ≤2.03)	3.75 (1.24, 11.37)	0.019	15.13(1.96, 116.92)	0.009
Baseline SII (>773.33 vs. ≤773.33)	3.58 (0.98, 13.13)	0.054		
Preoperative neutrophil (>2.805 vs. ≤2.805)	2.97 (0.82, 10.78)	0.099		
Preoperative lymphocyte (>1.175 vs. ≤1.175)	0.21 (0.05, 0.94)	0.041	0.06 (0.01, 0.63)	0.019
Preoperative dNLR (>-2.217 vs. ≤-2.217)	0.33 (0.09, 1.18)	0.088		

HR, hazard ratio; dNLR, derived neutrophil-to-lymphocyte ratio; SIRI, systemic inflammatory response index; SII, systemic immune-inflammation index.

### 3.5 Nomogram for predicting pathological response and DFS

**Figure 6:**
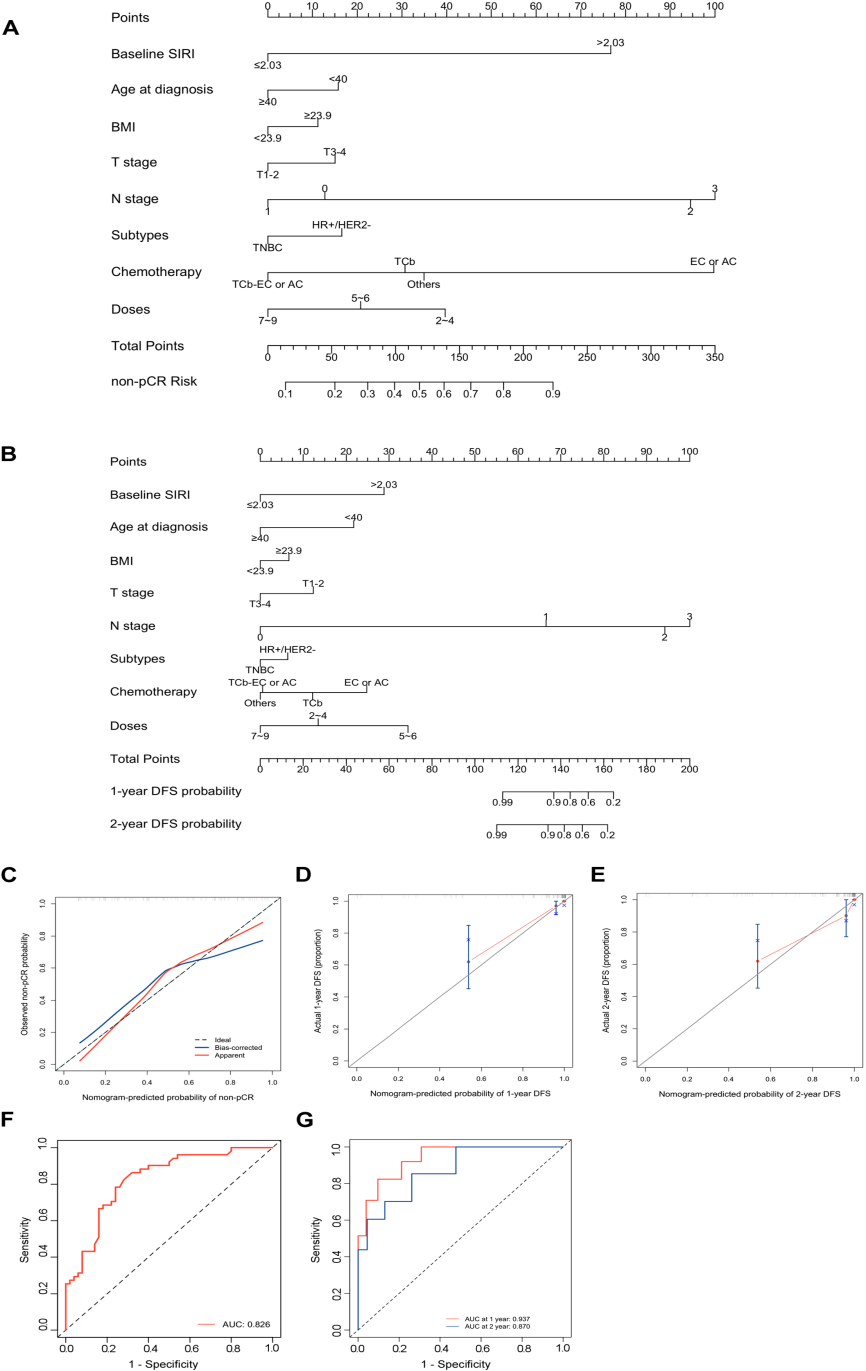
Development and performance of the nomograms for predicting pathological complete response (pCR) and Disease-Free Survival (DFS) in early-stage triple-negative breast cancer (eTNBC) patients following neoadjuvant chemoimmunotherapy (nCIT) **(A)** Nomogram for predicting the probability of pCR based on clinicopathological factors and systemic inflammatory response index (SIRI). **(B)** Nomogram for predicting the probability of DFS based on clinicopathological factors and SIRI. **(C–E)** Calibration plots for predicting pCR **(C)**, 1 year DFS **(D)**, 2 year DFS **(E)** in eTNBC patients after nCIT. **(F, G)** Receiver operating characteristic (ROC) curves of the nomogram for predicting pCR and DFS in eTNBC patients after nCIT.

**Figure 7:**
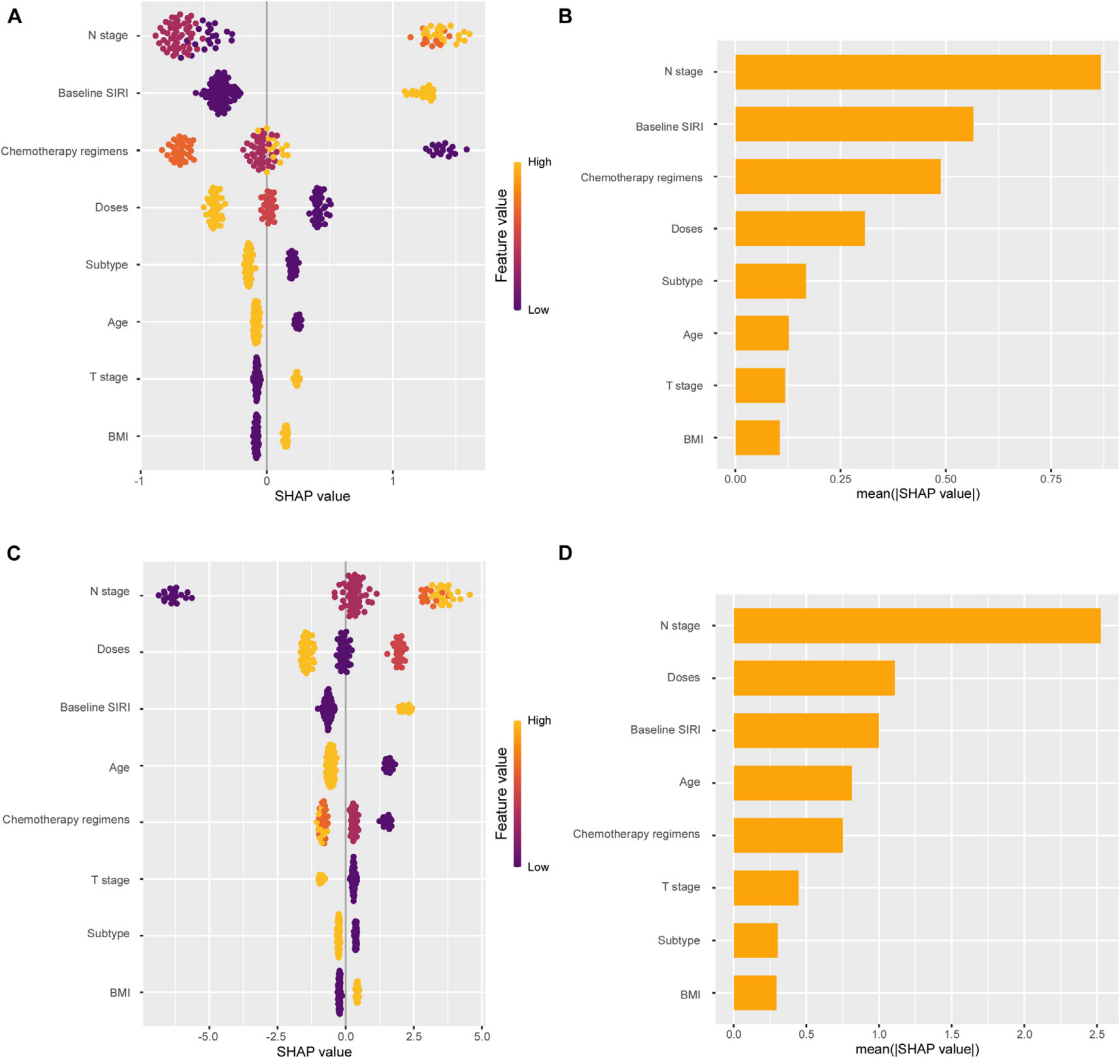
SHapley Additive exPlanations (SHAP) analysis showing feature importance for pathological complete response (pCR) and disease-free survival (DFS) prediction. **(A)** SHAP summary plot for pCR. **(B)** Mean SHAP values for each feature in pCR prediction. **(C)** SHAP summary plot for DFS. **(D)** Mean SHAP values for each feature in DFS prediction.

A nomogram was established to predict the probability of pCR and DFS for patients with eTNBC undergoing nCIT, based on significant risk factors and the independent predictive factor SIRI (**Figures 6A, B**). For predicting pCR probability and DFS, the nomograms achieved concordance index (C-index) were 0.826 (95% CI, 0.746~0.906) and 0.900 (95% CI, 0.820~0.980), respectively. Both models demonstrated good accuracy in predicting pCR and DFS, indicating reliable predictive performance. Calibration plots with bootstrap sampling (n=1000) were performed for each model, showing acceptable levels of agreement in the predicted outcomes (**Figures 6C–E**). Additionally, receiver operating characteristic (ROC) curve analysis revealed that the area under the curve (AUC) for predicting pCR was 0.826 (95% CI, 0.746~0.907), for 1-year DFS was 0.937 (95% CI, 0.872~1.000), and for 2-year DFS was 0.870 (95% CI, 0.737~1.000) (**Figures 6F, G**). To interpret the contributions of clinical features in predicting pCR and DFS, SHapley Additive exPlanations analysis was performed. In the model predicting pCR, N stage emerged as the variable exhibiting the highest contribution, followed sequentially by baseline SIRI, and chemotherapy regimens (**Figures 7A, B**). Lower N stage and lower baseline SIRI values were associated with a higher likelihood of achieving pCR. In the model predicting DFS, similar patterns were observed, N stage was the most significant contributor to the model, followed sequentially by doses of neoadjuvant immunotherapy, and baseline SIRI (**Figures 7C, D**). Higher values of these features correlated with shorter disease-free survival, highlighting their negative impact on long-term outcomes. These findings underscore the critical role of tumor burden and systemic inflammation in both treatment response and prognosis.

## 4 Discussion

For eTNBC, identifying effective biomarkers that can predict the efficacy of nCIT and forecast patient prognosis will help optimize precise and personalized treatment strategies, ultimately improving long-term outcomes (16). Primary biomarkers used to predict the efficacy of nCIT include PD-L1 expression, TILs, and tumor mutational burden (TMB). However, these biomarkers have several limitations, including high costs, invasive procedures, and significant variability in quantitative and diagnostic criteria for clinical utility. Additionally, TILs analysis is not routinely performed in clinical practice, as existing guidelines limit its utility (12–15). Therefore, there is a pressing need to develop simple, reliable, and cost-effective biomarkers to identify eTNBC patients likely to respond to nCIT therapy.

The predictive value of hematologic inflammatory markers have been demonstrated in nCIT pathological responses and treatment outcomes for non-small cell lung cancer (NSCLC) and esophageal cancer (30–34). Higher baseline NLR is associated with a lower likelihood of achieving pCR, while elevated preoperative NLR correlates with shorter disease-free survival (DFS) in NSCLC patients (30). This findings suggests that hematologic inflammatory markers play an important role in predicting the efficacy of nCIT in malignancies. However, the specific role of hematologic inflammatory markers in predicting efficacy in eTNBC with nCIT remains unproven. In this retrospective study, to the best of our knowledge, we present the comprehensive evaluation of the predictive role of inflammatory markers, including NLR, dNLR, PLR, SIRI, and SII, in predicting pCR and DFS in eTNBC patients treated with nCIT. Furthermore, we assessed clinicopathological data and morphological changes following treatment. Non-pCR group patients exhibited higher baseline levels of NLR, PLR, SIRI, and SII than pCR group. Elevated baseline lymphocyte counts were independently associated with higher pCR rates, whereas increased baseline monocytes, NLR, PLR, SIRI, SII, and preoperative SIRI were linked to lower pCR rates. Higher baseline SIRI was also associated with shorter DFS, while elevated preoperative lymphocytes correlated with better DFS. The total pCR rate in this study was 49.1%, lower than that reported in the KEYNOTE-522 trial, possibly due to differences regional population, clinical stage (36.6% *vs*. 24.7 in KEYNOTE-522) (8), individual heterogeneity and variations in the efficacy of difference ICIs. These results underscore the potential of hematologic inflammatory markers as valuable predictors of pathological response and prognosis in eTNBC patients undergoing nCIT.

Based on these findings, we developed two nomogram models incorporating the inflammatory marker SIRI to predict the probability of achieving pCR and DFS in eTNBC patients undergoing nCIT. These nomograms offer practical tools to potentially identify patients likely to benefit from treatment without requiring invasive biopsies or costly molecular tests. Our study provides evidence supporting the use of SIRI as a predictive marker for therapeutic response in eTNBC, addressing some limitations associated with static, tissue-based, and invasive assessments. Since SIRI utilizes routine blood parameters, it remains accessible even in resource-limited settings where tissue biomarker tests may not be feasible. These results suggest that clinical decisions regarding nCIT in eTNBC should comprehensively consider clinicopathological features, imaging characteristics, and baseline hematological indicators, particularly SIRI, given its potential influence on pCR and DFS outcomes. These findings suggest that the SIRI-based nomogram may offer a useful and clinically relevant tool for user-friendly risk stratification in this patient population. Nevertheless, external validation in multicenter cohorts is needed before clinical adoption, as our training data were retrospectively collected from a single institution.

Cancer-associated inflammation is a hallmark of malignancies, playing a crucial role in chemotherapy sensitivity, disease progression, and prognosis (20, 35–37). Systemic inflammatory responses are primarily driven by immune cells, cytokines, and soluble mediators (38, 39). Among emerging biomarkers, the SIRI has been identified as a reliable indicator of immune-inflammatory homeostasis and has gained increasing attention for its relevance in immunotherapy (40). Chronic inflammation fosters an immunosuppressive microenvironment by inducing immune exhaustion, thereby weakening antitumor responses (41, 42). Tumor-associated neutrophils contribute to immunotherapy resistance by promoting immunosuppression, disrupting antigen presentation, and inhibiting T and NK cell activation. Additionally, they facilitate immune evasion through ROS-induced DNA damage, leading to tumor antigen loss or mutation, and by modulating epigenetic pathways via exosomes (43–45). Monocytes also play a pivotal role in resistance, with a specific immunosuppressive subpopulation significantly expanded in treatment-resistant patients. This subset enhances immune evasion by inducing SOCS3 expression in CD4^+^ T cells (46). These findings underscore the critical role of inflammation in shaping the tumor immune landscape and influencing therapeutic outcomes.

Despite the valuable insights gained from this study, several limitations remain. First, as a retrospective study conducted at a single center, selection bias is inevitable. Additionally, the relatively small sample size and limited follow-up period may have constrained the ability to capture comprehensive long-term prognostic outcomes. Future investigations should focus on expanding the cohort and incorporating longer follow-up durations. Moreover, prospective and multicenter studies are essential to validate these findings and enhance their generalizability.

## 5 Conclusion

Hematologic inflammatory markers are cost-effective and convenient biomarkers for predicting the prognosis and treatment efficacy of eTNBC patients undergoing nCIT. The developed nomograms, incorporating the inflammatory marker SIRI, demonstrated high accuracy in estimating the probability of pCR and DFS in eTNBC patients treated with nCIT. These findings may assist clinicians in formulating personalized therapeutic strategies for eTNBC patients receiving nCIT.

## Data Availability

The raw data supporting the conclusions of this article will be made available by the authors, without undue reservation.

## References

[1] Bray F Laversanne M Sung H Ferlay J Siegel RL Soerjomataram I. Global cancer statistics 2022: GLOBOCAN estimates of incidence and mortality worldwide for 36 cancers in 185 countries. CA Cancer J Clin. (2024) 74:229–63. doi: 10.3322/caac.21834 38572751

[2] Loibl S Poortmans P Morrow M Denkert C Curigliano G. Breast cancer. Lancet. (2021) 397:1750–69. doi: 10.1016/S0140-6736(20)32381-3 33812473

[3] Zardavas D Piccart M. Neoadjuvant therapy for breast cancer. Annu Rev Med. (2015) 66:31–48. doi: 10.1146/annurev-med-051413-024741 25386936

[4] Cortazar P Zhang L Untch M Mehta K Costantino JP Wolmark N. Pathological complete response and long-term clinical benefit in breast cancer: the CTNeoBC pooled analysis. Lancet. (2014) 384:164–72. doi: 10.1016/S0140-6736(13)62422-8 24529560

[5] Spring LM Fell G Arfe A Sharma C Greenup R Reynolds KL. Pathologic complete response after neoadjuvant chemotherapy and impact on breast cancer recurrence and survival: A comprehensive meta-analysis. Clin Cancer Res. (2020) 26:2838–48. doi: 10.1158/1078-0432.CCR-19-3492 PMC7299787 32046998

[6] Symmans WF Yau C Chen YY Balassanian R Klein ME Pusztai L. Assessment of residual cancer burden and event-free survival in neoadjuvant treatment for high-risk breast cancer: an analysis of data from the I-SPY2 randomized clinical trial. JAMA Oncol. (2021) 7:1654–63. doi: 10.1001/jamaoncol.2021.3690 PMC8446908 34529000

[7] Liedtke C Mazouni C Hess KR Andre F Tordai A Mejia JA. Response to neoadjuvant therapy and long-term survival in patients with triple-negative breast cancer. J Clin Oncol. (2008) 26:1275–81. doi: 10.1200/JCO.2007.14.4147 18250347

[8] Schmid P Cortes J Pusztai L McArthur H Kummel S Bergh J. Pembrolizumab for early triple-negative breast cancer. N Engl J Med. (2020) 382:810–21. doi: 10.1056/NEJMoa1910549 32101663

[9] Schmid P Cortes J Dent R Pusztai L McArthur H Kummel S. Event-free survival with pembrolizumab in early triple-negative breast cancer. N Engl J Med. (2022) 386:556–67. doi: 10.1056/NEJMoa2112651 35139274

[10] Schmid P Cortes J Dent R McArthur H Pusztai L Kummel S. Overall survival with pembrolizumab in early-stage triple-negative breast cancer. N Engl J Med. (2024) 391:1981–91. doi: 10.1056/NEJMoa2409932 39282906

[11] Pusztai L Denkert C O’Shaughnessy J Cortes J Dent R McArthur H. Event-free survival by residual cancer burden with pembrolizumab in early-stage TNBC: exploratory analysis from KEYNOTE-522. Ann Oncol. (2024) 35:429–36. doi: 10.1016/j.annonc.2024.02.002 38369015

[12] Rayson VC Harris MA Savas P Hun ML Virassamy B Salgado R. The anti-cancer immune response in breast cancer: current and emerging biomarkers and treatments. Trends Cancer. (2024) 10:490–506. doi: 10.1016/j.trecan.2024.02.008 38521654

[13] Sompuram SR Torlakovic EE t Hart NA Vani K Bogen SA. Quantitative comparison of PD-L1 IHC assays against NIST standard reference material 1934. Mod Pathol. (2022) 35:326–32. doi: 10.1038/s41379-021-00884-w PMC8840973 34389791

[14] Wood SJ Gao Y Lee JH tHart NA Vani K Bogen SA. High tumor infiltrating lymphocytes are significantly associated with pathological complete response in triple negative breast cancer treated with neoadjuvant KEYNOTE-522 chemoimmunotherapy. Breast Cancer Res Treat. (2024) 205:193–9. doi: 10.1007/s10549-023-07233-2 38286889

[15] Isaacs J Anders C McArthur H Force J. Biomarkers of immune checkpoint blockade response in triple-negative breast cancer. Curr Treat Options Oncol. (2021) 22:38. doi: 10.1007/s11864-021-00833-4 33743085

[16] Villacampa G Navarro V Matikas A Ribeiro JM Schettini F Tolosa P. Neoadjuvant immune checkpoint inhibitors plus chemotherapy in early breast cancer: A systematic review and meta-analysis. JAMA Oncol. (2024) 10:1331–41. doi: 10.1001/jamaoncol.2024.3456 PMC12422158 39207778

[17] Crusz SM Balkwill FR. Inflammation and cancer: advances and new agents. Nat Rev Clin Oncol. (2015) 12:584–96. doi: 10.1038/nrclinonc.2015.105 26122183

[18] Walther KA Groger S Vogler JAH Wostmann B Meyle J. Inflammation indices in association with periodontitis and cancer. Periodontol 2000. (2024) 96:281–315. doi: 10.1111/prd.12612 39317462 PMC11579835

[19] Dowling GP Daly GR Hegarty A Hembrecht S Bracken A Toomey S. Predictive value of pretreatment circulating inflammatory response markers in the neoadjuvant treatment of breast cancer: meta-analysis. Br J Surg. (2024) 111. doi: 10.1093/bjs/znae132 PMC11129713 38801441

[20] Dong J Sun Q Pan Y Lu N Han X Zhou Q. Pretreatment systemic inflammation response index is predictive of pathological complete response in patients with breast cancer receiving neoadjuvant chemotherapy. BMC Cancer. (2021) 21:700. doi: 10.1186/s12885-021-08458-4 34126950 PMC8204500

[21] Zhang S Cheng T. Prognostic and clinicopathological value of systemic inflammation response index (SIRI) in patients with breast cancer: a meta-analysis. Ann Med. (2024) 56:2337729. doi: 10.1080/07853890.2024.2337729 38569199 PMC10993763

[22] Huang W Xiong Z Zhong W Zhang C Feng J Wang X. Development of a nomogram for predicting survival of breast cancer patients with neoadjuvant chemotherapy: a dynamic analysis for systemic inflammation response index. Gland Surg. (2023) 12:1459–74. doi: 10.21037/gs-23-226 PMC10721566 38107499

[23] Chen L Kong X Wang Z Wang X Fang Y Wang J. Pre-treatment systemic immune-inflammation index is a useful prognostic indicator in patients with breast cancer undergoing neoadjuvant chemotherapy. J Cell Mol Med. (2020) 24:2993–3021. doi: 10.1111/jcmm.14934 31989747 PMC7077539

[24] Ocana A Chacon JI Calvo L Anton A Mansutti M Albanell J. Derived neutrophil-to-lymphocyte ratio predicts pathological complete response to neoadjuvant chemotherapy in breast cancer. Front Oncol. (2021) 11:827625. doi: 10.3389/fonc.2021.827625 35223459 PMC8875201

[25] Graziano V Grassadonia A Iezzi L Vici P Pizzuti L Barba M. Combination of peripheral neutrophil-to-lymphocyte ratio and platelet-to-lymphocyte ratio is predictive of pathological complete response after neoadjuvant chemotherapy in breast cancer patients. Breast. (2019) 44:33–8. doi: 10.1016/j.breast.2018.12.014 30611095

[26] Ogston KN Miller ID Payne S Hutcheon AW Sarkar TK Smith I. A new histological grading system to assess response of breast cancers to primary chemotherapy: prognostic significance and survival. Breast. (2003) 12:320–7. doi: 10.1016/s0960-9776(03)00106-1 14659147

[27] Wolff AC Hammond MEH Allison KH Harvey BE Mangu PB Bartlett JMS. Human epidermal growth factor receptor 2 testing in breast cancer: american society of clinical oncology/college of american pathologists clinical practice guideline focused update. Arch Pathol Lab Med. (2018) 142:1364–82. doi: 10.5858/arpa.2018-0902-SA 29846104

[28] Nielsen TO Leung SCY Rimm DL Dodson A Acs B Badve S. Assessment of ki67 in breast cancer: updated recommendations from the international ki67 in breast cancer working group. J Natl Cancer Inst. (2021) 113:808–19. doi: 10.1093/jnci/djaa201 PMC8487652 33369635

[29] Loibl S André F Bachelot T Barrios CH Bergh J Burstein HJ. Early breast cancer: ESMO Clinical Practice Guideline for diagnosis, treatment and follow-up. Ann Oncol. (2024) 35:159–82. doi: 10.1016/j.annonc.2023.11.016 38101773

[30] Liu W Ren S Yang L Xiao Y Zeng C Chen C. The predictive role of hematologic markers in resectable NSCLC patients treated with neoadjuvant chemoimmunotherapy: a retrospective cohort study. Int J Surg. (2023) 109:3519–26. doi: 10.1097/JS9.0000000000000650 PMC10651234 37578441

[31] Li C Wu J Jiang L Zhang L Huang J Tian Y. The predictive value of inflammatory biomarkers for major pathological response in non-small cell lung cancer patients receiving neoadjuvant chemoimmunotherapy and its association with the immune-related tumor microenvironment: a multi-center study. Cancer Immunol Immunother. (2023) 72:783–94. doi: 10.1007/s00262-022-03262-w PMC10991885 36056951

[32] Huai Q Luo C Song P Bie F Bai G Li Y. Peripheral blood inflammatory biomarkers dynamics reflect treatment response and predict prognosis in non-small cell lung cancer patients with neoadjuvant immunotherapy. Cancer Sci. (2023) 114:4484–98. doi: 10.1111/cas.15964 PMC10728017 37731264

[33] Xu F Zhu H Dong Y Li L Liu N Yuan S. Combined inflammatory parameters and tertiary lymphoid structure predict prognosis in patients with resectable non-small cell lung cancer treated with neoadjuvant chemoimmunotherapy. Front Immunol. (2023) 14:1244256. doi: 10.3389/fimmu.2023.1244256 38155965 PMC10752966

[34] Han W Weng K Zhang P Hong Z. Predictive value of systemic immune-inflammation index for pathological complete response in patients receiving neoadjuvant immunochemotherapy for locally advanced esophageal cancer. Front Surg. (2022) 9:1091601. doi: 10.3389/fsurg.2022.1091601 36684142 PMC9845901

[35] Hanahan D. Hallmarks of cancer: new dimensions. Cancer discovery. (2022) 12:31–46. doi: 10.1158/2159-8290.Cd-21-1059 35022204

[36] Zhu M Chen L Kong X Wang X Fang Y Li X. The systemic inflammation response index as an independent predictor of survival in breast cancer patients: A retrospective study. Front Mol biosciences. (2022) 9:856064. doi: 10.3389/fmolb.2022.856064 PMC8918696 35295846

[37] Tang C Zhang M Jia H Wang T Wu H Xu K. The systemic inflammation response index (SIRI) predicts survival in advanced non-small cell lung cancer patients undergoing immunotherapy and the construction of a nomogram model. Front Immunol. (2024) 15:1516737. doi: 10.3389/fimmu.2024.1516737 39776905 PMC11703897

[38] Qi Q Zhuang L Shen Y Geng Y Yu S Chen H. A novel systemic inflammation response index (SIRI) for predicting the survival of patients with pancreatic cancer after chemotherapy. Cancer. (2016) 122:2158–67. doi: 10.1002/cncr.30057 27152949

[39] Diakos CI Charles KA McMillan DC Clarke SJ. Cancer-related inflammation and treatment effectiveness. Lancet Oncology. (2014) 15:e493–503. doi: 10.1016/s1470-2045(14)70263-3 25281468

[40] Yan D Dai C Xu R Huang Q Ren W. Predictive ability of systemic inflammation response index for the risk of pneumonia in patients with acute ischemic stroke. Gerontology. (2023) 69:181–8. doi: 10.1159/000524759 35584610

[41] Shim R Wong CH. Ischemia, immunosuppression and infection–tackling the predicaments of post-stroke complications. Int J Mol Sci. (2016) 17. doi: 10.3390/ijms17010064 PMC4730309 26742037

[42] Liu DD Chu SF Chen C Yang PF Chen NH He X. Research progress in stroke-induced immunodepression syndrome (SIDS) and stroke-associated pneumonia (SAP). Neurochemistry Int. (2018) 114:42–54. doi: 10.1016/j.neuint.2018.01.002 29317279

[43] Yao J Ji L Wang G Ding J. Effect of neutrophils on tumor immunity and immunotherapy resistance with underlying mechanisms. Cancer Commun (London England). (2025) 45:15–42. doi: 10.1002/cac2.12613 PMC11758154 39485719

[44] Cadet J Wagner JR. DNA base damage by reactive oxygen species, oxidizing agents, and UV radiation. Cold Spring Harbor Perspect Biol. (2013) 5. doi: 10.1101/cshperspect.a012559 PMC3552502 23378590

[45] Kornepati AVR Rogers CM Sung P Curiel TJ. The complementarity of DDR, nucleic acids and anti-tumour immunity. Nature. (2023) 619:475–86. doi: 10.1038/s41586-023-06069-6 37468584

[46] Keenan BP McCarthy EE Ilano A Yang H Zhang L Allaire K. Circulating monocytes associated with anti-PD-1 resistance in human biliary cancer induce T cell paralysis. Cell reports. (2022) 40:111384. doi: 10.1016/j.celrep.2022.111384 36130508 PMC10060099

