# Supplementary material for: Pathological complete response and prognostic predictive factors of neoadjuvant chemoimmunotherapy in early stage triple-negative breast cancer
Source: Front Immunol. 2025 May 12;16:1570394. doi: 10.3389/fimmu.2025.1570394 (PMC12104239; doi:10.3389/fimmu.2025.1570394)
Supplement: Supplementary file 1 [file Table1.docx]

Table S1 ROC cut-off value of hematologic-related inflammation markers

| Hematological parameters | ROC cut-off value |
| --- | --- |
| Baseline platelet | 213 |
| Baseline neutrophil | 6.015 |
| Baseline lymphocyte | 1.165 |
| Baseline monocyte | 0.31 |
| Baseline NLR | 2.71 |
| Baseline dNLR | -5.26 |
| Baseline PLR | 140.24 |
| Baseline SIRI | 2.03 |
| Baseline SII | 773.33 |
| Preoperative platelet | 198 |
| Preoperative neutrophil | 2.805 |
| Preoperative lymphocyte | 1.175 |
| Preoperative monocyte | 0.485 |
| Preoperative NLR | 1.913 |
| Preoperative dNLR | -2.217 |
| Preoperative PLR | 242.118 |
| Preoperative SIRI | 0.403 |
| Preoperative SII | 608.345 |

NLR,neutrophilto-lymphocyte ratio; dNLR, derived neutrophil-to-lymphocyte ratio; PLR, platelet-to-lymphocyte ratio; SIRI, systemic inflammatory response index; SII, systemic immune-inflammation index.
