# Supplementary material for: Pathological complete response and prognostic predictive factors of neoadjuvant chemoimmunotherapy in early stage triple-negative breast cancer
Source: Front Immunol. 2025 May 12;16:1570394. doi: 10.3389/fimmu.2025.1570394 (PMC12104239; doi:10.3389/fimmu.2025.1570394)
Supplement: Supplementary file 2 [file Table2.docx]

Table S2 Clinicopathological data of 13 progression cases

| **No.** | **Age** | **Clinical**  **stage** | **Pathological response after nCIT** | **Lymphovascular invasion** | **Miller-payne grade** | **Progression time interval** | **Type of progression after nCIT** |
| --- | --- | --- | --- | --- | --- | --- | --- |
| 1 | 41 | IIIC | PR | No | 5 | 11 | Liver metastasis |
| 2 | 55 | IIIC | PR | Yes | 3 | 10 | Bone metastasis |
| 3 | 32 | IIIA | PR | Yes | 3 | 3 | chest wall recurrence |
| 4 | 64 | IIIA | PR | Yes | 2 | 7 | contralateral upper arm skin metastasis |
| 5 | 39 | IIIA | PR | Yes | 2 | 9 | chest wall recurrence |
| 6 | 64 | IIIA | PR | Yes | 2 | 8 | Liver metastasis |
| 7 | 30 | IIIC | PR | Yes | 2 | 41 | chest wall recurrence |
| 8 | 30 | IIB | PR | No | 3 | 11 | Lung metastasis |
| 9 | 47 | IIIC | SD | Yes | 2 | 3 | Bone metastasis |
| 10 | 40 | IIIA | SD | No | 2 | 17 | Lung metastasis |
| 11 | 52 | IIB | SD | No | 1 | 4 | Lung metastasis |
| 12 | 38 | IIIA | SD | Yes | 1 | 2 | Liver metastasis |
| 13 | 55 | IIIC | PD | No | 1 | 6 | Liver metastasis |

nCIT, neoadjuvant chemoimmunotherapy; PR, partial response; SD, stable disease; PD, progressive disease.
