# Supplementary material for: Pathological complete response and prognostic predictive factors of neoadjuvant chemoimmunotherapy in early stage triple-negative breast cancer
Source: Front Immunol. 2025 May 12;16:1570394. doi: 10.3389/fimmu.2025.1570394 (PMC12104239; doi:10.3389/fimmu.2025.1570394)
Supplement: Supplementary file 3 [file Table3.docx]

Table S3 Hematological parameters of the patients

| **Characteristics** | **Total (n=112)** | **pCR (n=55)** | **Non-pCR (n=57)** | ***P*** |
| --- | --- | --- | --- | --- |
| Baseline platelet | 270.50 (225.00-320.00) | 268.00 (197.00-323.00) | 272.00 (232.00-317.00) | 0.214 |
| Baseline neutrophil | 4.12 (3.32-5.25) | 3.98 (3.15-4.95) | 4.50 (3.40-6.07) | 0.060 |
| Baseline lymphocyte | 1.55 (1.29-1.98) | 1.58 (1.38-2.02) | 1.52 (1.13-1.87) | 0.057 |
| Baseline monocyte | 0.39 (0.29-0.57) | 0.36 (0.27-0.50) | 0.42 (0.33-0.61) | 0.038 |
| Baseline NLR | 2.72 (1.89-3.94) | 2.40 (1.68-3.32) | 3.22 (2.02-4.79) | 0.001 |
| Baseline dNLR | -3.46 (-4.57~-2.67) | -3.30 (-4.25~-2.61) | -3.82(-5.30~-2.79) | 0.073 |
| Baseline PLR | 167.71 (127.14-223.87) | 147.49 (121.76-196.14) | 182.57 (151.95-255.42) | 0.001 |
| Baseline SIRI | 1.03 (0.64-1.65) | 0.88 (0.54-1.30) | 1.18 (0.76-2.88) | 0.003 |
| Baseline SII | 755.93 (480.70-1049.53) | 615.01 (382.37-879.52) | 882.20 (623.31-1261.51) | <0.001 |
| Preoperative platelet | 189.50 (157.25-250.00) | 183.00 (157.00-259.00) | 199.00 (157.50-250.00) | 0.978 |
| Preoperative neutrophil | 2.97 (2.11-4.19) | 2.77 (2.07-4.00) | 3.07 (2.13-4.30) | 0.505 |
| Preoperative lymphocyte | 1.12 (0.95-1.38) | 1.22 (0.95-1.39) | 1.06 (0.94-1.34) | 0.449 |
| Preoperative monocyte | 0.38 (0.29-0.51) | 0.38 (0.30-0.48) | 0.38 (0.29-0.53) | 0.753 |
| Preoperative NLR | 2.89 (1.76-4.06) | 2.68 (1.54-3.98) | 3.07 (2.00-4.42) | 0.465 |
| Preoperative dNLR | -2.26 (-3.43~-1.56) | -2.13 (-3.35~-1.58) | -2.37 (-3.57~-1.53) | 0.539 |
| Preoperative PLR | 164.56 (120.60-239.21) | 159.74 (126.73-206.52) | 171.58 (117.88-254.54) | 0.725 |
| Preoperative SIRI | 0.96 (0.51-1.92) | 0.94 (0.40-1.89) | 0.99 (0.59-1.94) | 0.451 |
| Preoperative SII | 539.83 (249.19-924.90) | 508.76 (237.98-952.07) | 582.18 (282.86-923.63) | 0.665 |

NLR, neutrophilto-lymphocyte ratio; dNLR, derived neutrophil-to-lymphocyte ratio; PLR, platelet-to-lymphocyte ratio; SIRI, systemic inflammatory response index; SII, systemic immune-inflammation index.
