# Supplementary material for: Pathological complete response and prognostic predictive factors of neoadjuvant chemoimmunotherapy in early stage triple-negative breast cancer
Source: Front Immunol. 2025 May 12;16:1570394. doi: 10.3389/fimmu.2025.1570394 (PMC12104239; doi:10.3389/fimmu.2025.1570394)
Supplement: Supplementary file 4 [file Table4.docx]

| **Table S4 Univariate and multivariate analyses for non-pCR** | | | | | | | |
| --- | --- | --- | --- | --- | --- | --- | --- |
| **Characteristics** | **Univariate analysis** | | |  | **Multivariate analysis** | | |
|  | **OR (95%CI)** |  | ***P*** |  | **OR (95%CI)** |  | ***P*** |
| Age (≥40 vs. <40) (year) | 0.81 (0.35, 1.90) |  | 0.813 |  |  |  |  |
| BMI, kg/m^2^ (>23.9 vs. ≤23.9) | 1.40 (0.63, 3.14) |  | 0.413 |  |  |  |  |
| Menopausal status (post-menopausal vs. pre-menopausal) | 1.56 (0.70, 3.47) |  | 0.280 |  |  |  |  |
| Family history (Yes vs. No) | 1.20 (0.47, 3.06) |  | 0.702 |  |  |  |  |
| N grade |  |  |  |  |  |  |  |
| N1 vs. N0 | 0.57 (0.20, 1.59) |  | 0.280 |  |  |  |  |
| N2 vs. N0 | 5.50 (0.96, 31.43) |  | 0.055 |  |  |  |  |
| N3 vs. N0 | 2.50 (0.69, 9.08) |  | 0.164 |  |  |  |  |
| Ki-67 index (>30% vs. ≤30%) | 0.41 (0.12, 1.42) |  | 0.159 |  |  |  |  |
| Surgery type (Breast conserving surgery vs. Mastectomy ) | 1.46 (0.58, 3.66) |  | 0.426 |  |  |  |  |
| Lymph node dissection (ALND vs. SLNB) | 0.41 (0.10, 1.68) |  | 0.217 |  |  |  |  |
| Preoperative platelet (>198 vs. ≤198) | 1.44 (0.68, 3.04) |  | 0.337 |  |  |  |  |
| Preoperative neutrophil (>2.805 vs. ≤2.805) | 1.91 (0.90, 4.05) |  | 0.092 |  |  |  |  |
| Preoperative monocyte (>0.485 vs. ≤0.485) | 1.89 (0.83, 4.29) |  | 0.131 |  |  |  |  |
| Preoperative NLR (>1.913 vs. ≤1.913) | 2.14 (0.92, 4.97) |  | 0.076 |  |  |  |  |
| Preoperative dNLR (>-2.217 vs. ≤-2.217) | 0.56 (0.27, 1.19) |  | 0.134 |  |  |  |  |
| Preoperative SII (>608.345 vs. ≤608.345) | 1.56 (0.74, 3.32) |  | 0.244 |  |  |  |  |

OR, odds ratio; SLNB, sentinel lymph node biopsy; ALND, axillary lymph node dissection; NLR, neutrophilto-lymphocyte ratio; dNLR, derived neutrophil-to-lymphocyte ratio; SII, systemic immune-inflammation index.
