# Supplementary material for: Pathological complete response and prognostic predictive factors of neoadjuvant chemoimmunotherapy in early stage triple-negative breast cancer
Source: Front Immunol. 2025 May 12;16:1570394. doi: 10.3389/fimmu.2025.1570394 (PMC12104239; doi:10.3389/fimmu.2025.1570394)
Supplement: Supplementary file 5 [file Table5.docx]

| **Characteristics** | **Univariate analysis** | | |  | **Multivariate analysis** | | |
| --- | --- | --- | --- | --- | --- | --- | --- |
|  | **HR(95% CI)** |  | ***P* value** |  | **HR(95% CI)** |  | ***P* value** |
| Age (≥40 vs. <40) (year) | 0.48 (0.16, 1.47) |  | 0.198 |  |  |  |  |
| BMI, kg/m^2^ (>23.9 vs. ≤23.9) | 1.80 (0.60, 5.39) |  | 0.291 |  |  |  |  |
| Menopausal status (post-menopausal vs. pre-menopausal) | 1.41 (0.46, 4.32) |  | 0.550 |  |  |  |  |
| Family history (Yes vs. No) | 0.30 (0.04, 2.33) |  | 0.251 |  |  |  |  |
| Doses of nCIT |  |  |  |  |  |  |  |
| 5~6 vs. 2~4 | 1.04 (0.30, 3.70) |  | 0.952 |  |  |  |  |
| 7~9 vs. 2~4 | 0.32 (0.06, 1.56) |  | 0.158 |  |  |  |  |
| Baseline platelet (>213 vs. ≤213) | 3.54 (0.46, 27.25) |  | 0.225 |  |  |  |  |
| Baseline lymphocyte (>1.165 vs. ≤1.165) | 0.48 (0.15, 1.56) |  | 0.222 |  |  |  |  |
| Baseline monocyte (>0.31 vs. ≤0.31) | 2.24 (0.49, 10.22) |  | 0.298 |  |  |  |  |
| Baseline NLR (>2.71 vs. ≤2.71) | 2.73 (0.74, 10.09) |  | 0.132 |  |  |  |  |
| Baseline PLR (>140.24 vs. ≤140.24) | 2.77 (0.61, 12.51) |  | 0.185 |  |  |  |  |
| Preoperative platelet (>198 vs. ≤198) | 1.01 (0.34, 3.00) |  | 0.990 |  |  |  |  |
| Preoperative monocyte (>0.485 vs. ≤0.485) | 1.54 (0.50, 4.73) |  | 0.449 |  |  |  |  |
| Preoperative NLR (>1.913 vs. ≤1.913) | 1.92 (0.42, 8.79) |  | 0.398 |  |  |  |  |
| Preoperative PLR (>242.118 vs. ≤242.118) | 2.40 (0.78, 7.36) |  | 0.126 |  |  |  |  |
| Preoperative SIRI (>0.403 vs. ≤0.403) | 2.29 (0.30, 17.72) |  | 0.428 |  |  |  |  |
| Preoperative SII (>608.345 vs. ≤608.345) | 2.01 (0.66, 6.17) |  | 0.507 |  |  |  |  |

Table S5 Univariate and multivariate analyses for DFS

HR, hazard ratio; NLR, neutrophilto-lymphocyte ratio; PLR, platelet-to-lymphocyte ratio; SIRI, systemic inflammatory response index; SII, systemic immune-inflammation index.
